# Effect of air sterilizers in an outpatient clinic at a tertiary university hospital

**DOI:** 10.3389/fmed.2024.1375260

**Published:** 2024-04-04

**Authors:** Dong Hoon Lee, Sujung Yeom, Hwa Sin Lee, Hyong-Ho Cho

**Affiliations:** ^1^Department of Otolaryngology-Head and Neck Surgery, Chonnam National University Medical School, Chonnam National University Hwasun Hospital, Gwangju, Republic of Korea; ^2^Chonnam National University Hospital, Gwangju, Republic of Korea

**Keywords:** air, air filters, microorganisms, volatile organic compounds, hospitals

## Abstract

**Background:**

After the COVID-19 outbreak, interest in airborne virus infections has increased. We considered ways to reduce the risk of infection to other people by inactivating the virus before it is inhaled into the heating, ventilation, and air conditioning (HVAC) systems. We installed a recently developed air sterilizer in the newly remodeled outpatient clinic of a tertiary university hospital and confirmed its effectiveness.

**Methods:**

After remodeling the ENT outpatient clinic at Chonnam National University Hospital, 15 KOKKOS air sterilizers (Bentech Frontier Co., Ltd., Gwangju, Korea) were installed. Total culturable microorganisms (TCMs) and volatile organic compounds (VOCs) were measured in five separate inspection areas three days before installation, 2 weeks after installation, and 4 weeks after installation.

**Results:**

After measurement of TCMs, improvement in air quality occurred 2 weeks after air sterilizer instatement at all timepoints except inspection area 5, and further improvement was achieved after 4 weeks (*p* < 0.05). After assessment of VOCs, improvement occurred 4 weeks after air sterilizer connection at all points (*p* < 0.05).

**Conclusion:**

KOKKOS air sterilizers are effective in improving air quality in an outpatient clinic at a tertiary university hospital.

## Introduction

After the COVID-19 outbreak, interest in airborne virus infections has increased (1–8). Natural ventilation is difficult in hospitals due to patient overcrowding, and mechanical sources have been applied to assist in ventilation (1–8). Therefore, heating, ventilation, and air conditioning (HVAC) systems are beginning to be studied more deeply to help eliminate airborne viruses, fungi, and bacteria (1–3, 7, 8).

However, when the Middle East Respiratory Syndrome (MERS) spread, infection within buildings through HVAC systems was confirmed (9). Therefore, the authors considered ways to reduce the risk of infection to other people by inactivating the virus before it is inhaled into the HVAC system (2, 3). We installed a recently developed air sterilizer

in the newly remodeled outpatient clinic of a tertiary university hospital and confirmed its effectiveness.

## Materials and methods

**FIGURE 1:**
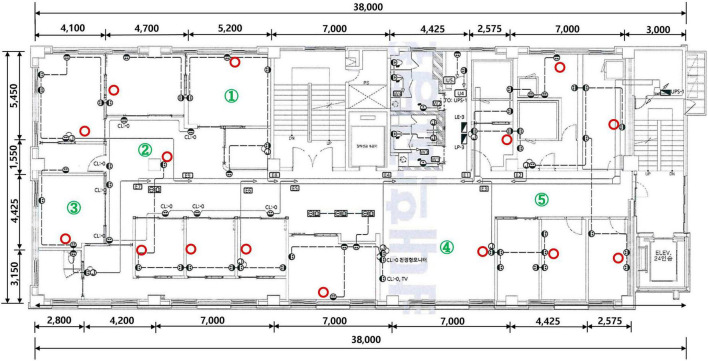
KOKKOS (Bentech Frontier Co., Ltd., Gwangju, Korea) air sterilizer installation location (red circle) and air quality measurement stations (numbers in green circle).

This study was conducted with the approval of the Institutional Review Board of Chonnam National University Hwasun Hospital. After remodeling the ENT outpatient clinic at Chonnam National University Hospital, 15 KOKKOS air sterilizers (Bentech Frontier Co., Ltd., Gwangju, Korea) were installed based on the clean air delivery rate (CADR) value (Figure 1). KOKKOS air sterilizers is equipped with a VORTEX function on the front air intake part to enhance indoor air intake with a suction flow speed of up to 4 m/s or more. It was independently fixed to a wall 1.5 m above the ground in order to absorb the air released when a person breathes indoors as quickly as possible.

**FIGURE 2:**
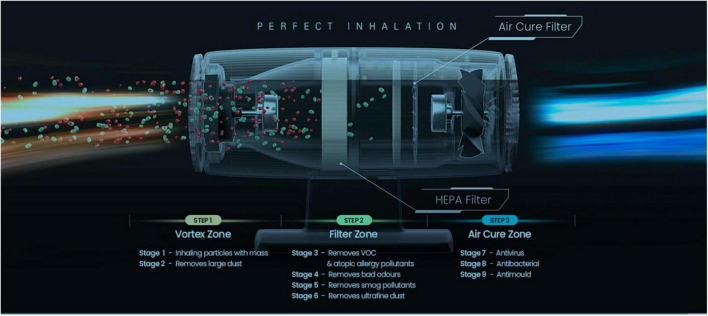
Description of internal functionality of KOKKOS (Bentech Frontier Co., Ltd., Gwangju, Korea).

KOKKOS air sterilizers contains two filters, and the HEPA Filter at the front primarily filters PM1.0-size fine dust, bacteria, and viruses, and the sterilization filter at the rear plays a major role in inactivating viruses by generating OH-radicals. KOKKOS sterilization filters were also applied to rapidly and completely eliminate harmful substances such as volatile organic compounds (VOCs), bad smells, harmful gases, bacteria, and viruses without discharging them. Additionally, the KOKKOS filter also included a HEPA filter to remove fine particulate matter (Figure 2). In recognition of its excellent infection prevention, KOKKOS was designated as one of Top 100 National R&D Technologies with Outstanding Performance in 2020.

To analyze the impact of KOKKOS on the indoor air quality of the newly remodeled ENT outpatient clinic, total culturable microorganisms (TCMs) and VOCs were measured three days before installation, 2 weeks after installation, and 4 weeks after installation of the air sterilizers. Because the ENT outpatient clinic is divided into several structures, including a treatment room, waiting room, and examination room, we conducted air quality tests in five different locations to ensure the accuracy of the test (Figure 1). TCMs was measured with KAS-110 (1CFM, 28.3 L/m) equipment using the “Indoor Air Quality Test Method” (10). After installing the bacteria and fungi culture medium on the equipment, TCMs was measured three times at each of five designated locations. VOCs were measured with GC Mass equipment using the “Indoor Air Quality Test Method” (10). VOCs were measured three times at each of the five designated locations.

All statistical calculations were performed using SPSS version 28.0. Concentration differences and measurement timing as dependent variables were examined by the Friedman test and the Wilcoxon signed rank test. In all analyses, *p* < 0.05 was considered statistically significant.

## Results

The results of TCMs and VOCs conducted three days before installation, 2 weeks after installation, and 4 weeks after installation are summarized in Tables 1, 2.

**TABLE 1:** Total culturable microorganism concentrations (CFU/m^3^) before and after air sterilizer installation.

Measurement location	3 days before installation	2 weeks after installation	4 weeks after installation	Improvement rate (%)
Inspection area 1	73	33	18	75.3
Inspection area 2	98	49	24	75.5
Inspection area 3	62	24	12	80.6
Inspection area 4	159	103	50	68.6
Inspection area 5	104	124	62	40.4
Average	99.2	66.6	33.2	68.1

**TABLE 2:** Total volatile organic compound concentrations (μg/m^3^) before and after air sterilizer installation.

Measurement location	3 days before installation	2 weeks after installation	4 weeks after installation	Improvement rate (%)
Inspection area 1	46.7	46.1	33.9	27.4
Inspection area 2	52.2	58.6	38.8	25.7
Inspection area 3	349.7	87.1	18.8	94.6
Inspection area 4	61.7	76.7	37.7	38.9
Inspection area 5	61.4	73.8	50.2	18.2
Average	114.34	68.5	35.9	41.0

**FIGURE 3:**
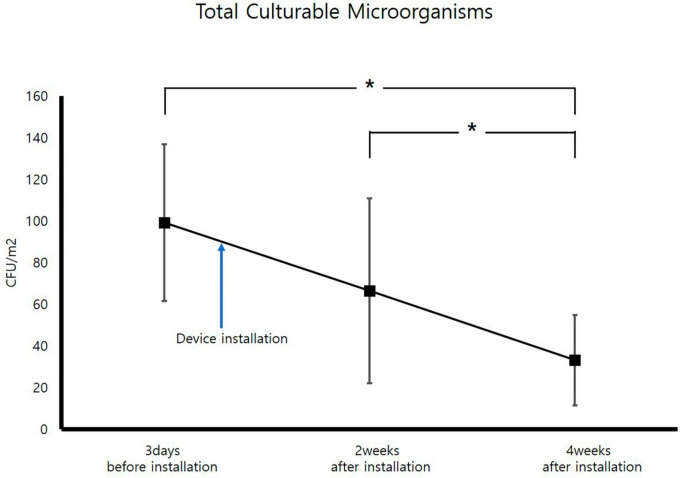
Change in average total culturable microorganism concentrations (CFU; Colony forming unit, *p < 0.05).

For measurement of TCMs, improvement occurred 2 weeks after air sterilizer installation at all points except inspection area 5, and further improvement was achieved after 4 weeks (*p* < 0.05). The area with the highest TCMs improvement rate was inspection area 3, which displayed an enhancement rate of 80.6%. The area with the lowest improvement rate was inspection area 5 (40.4%). The average improvement rate for removal of TCM for the entire space was 68.1%, confirming that the air sterilizer was effective (Figure 3).

**FIGURE 4:**
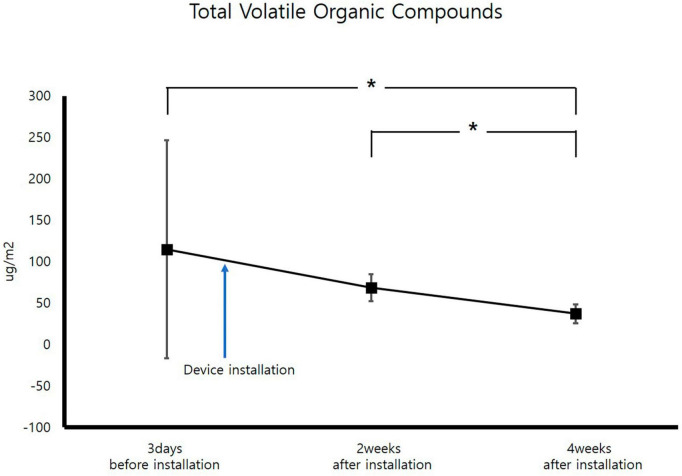
Change in average total volatile organic compound concentrations (*p < 0.05).

After VOCs assessment, improvement occurred 4 weeks after air sterilizer installation at all points (*p* < 0.05). The areas with the highest and lowest VOCs improvement rate were inspection area 3 and 5, which demonstrated improvement rates of 94.6 and 18.2%, respectively. The average improvement rate of VOCs for the entire space was 41.0%, verifying the efficacy of the air sterilizer (Figure 4).

## Discussion

After comparing TCMs and VOCs before and after installation of the KOKKOS air sterilizers, air quality improvement was evident (*p* < 0.05). ENT outpatient clinics have many patients with colds accompanied by coughand aerosol generation, therefore, the risk of nosocomial infection is high (5, 7). In this situation, KOKKOS air sterilizers may play a significant role in prevention of hospital-acquired infections.

Following the measurement of TCM concentrations prior to installion of the air sterilizers, the maximum value was 159 CFU/m^3^, which is significantly lower than the air quality management standard of 800 CFU/m^3^ for multi-use facilities in South Korea, confirming that air quality is being managed well. TCMs concentrations exhibited a statistically significant improvement in air quality before and 4 weeks after installation of air sterilizers (*p* < 0.05). Additionally, TCMs concentrations revealed a statistically significant improvement in air quality 4 weeks after instatement of the air sterilizers compared to 2 weeks after installation (*p* < 0.05).

Assessment of VOCs concentrations before installation of the air sterilizer disclosed almost similar VOC concentrations across all areas except for inspection area 3. For inspection area 3, the concentration was judged to be high due to chemicals used inside. The improvement rate was considered low due to the low VOCs concentrations, but air quality showed a statistically significant improvement in VOCs concentrations before and 4 weeks after instatement of the air sterilizer (*p* < 0.05). Additionally, VOCs concentration showed a statistically significant improvement in air quality 4 weeks after installation of the air sterilizer as compared to 2 weeks after installation (*p* < 0.05).

Inspection area 3 exhibited the greatest improvement in both TCMs (80.6%) and VOCs (94.6%) after air sterilizer installation. This effect is believed to be because this is where most aerosol-generating procedures, such as local anesthesia surgery and endoscopic procedures, are performed among outpatient treatment spaces.

On the other hand, in inspection area 5, both TCMs (40.4%) and VOCs (18.2%) had the least improvement after air sterilizer installation, because this was a corridor space with a lot of movement and strong air flow. As mentioned above, TCMs and VOCs in an ENT outpatient clinic were statistically significantly improved after installing KOKKOS air sterilizers.

## Conclusion

KOKKOS air sterilizers significantly reduce the occurrence of TCMs and VOCs in an ENT outpatient clinic. The installation of KOKKOS air sterilizers is expected to diminish the existence of TCMs and VOCs, to help prevent hospital-acquired infections, and to protect the health of medical staff and patients.

## Data availability statement

The original contributions presented in the study are included in the article/Supplementary material, further inquiries can be directed to the corresponding author.

## Author contributions

DL: Writing−original draft, Writing−review and editing. SY: Writing−review and editing. HL: Writing−review and editing. H-HC: Writing−original draft, Writing−review and editing.
